# Evaluation of MR-safe bioptomes for MR-guided endomyocardial biopsy in minipigs: a potential radiation-free clinical approach

**DOI:** 10.1186/s41747-023-00391-4

**Published:** 2023-12-05

**Authors:** Angelika Svetlove, Christian O. Ritter, Christian Dullin, Michael Schmid, Senta Schauer, Johannes Uihlein, Martin Uecker, Matthias Mietsch, Christine Stadelmann, Joachim Lotz, Christina Unterberg-Buchwald

**Affiliations:** 1 Translational Molecular Imaging, Max Planck Institute for Multidisciplinary Sciences, Göttingen, Germany; 2 Cluster of Excellence “Multiscale Bioimaging: From Molecular Machines to Networks of Excitable Cells” (MBExC), University of Göttingen, Göttingen, Germany; 3 Institute for Diagnostic and Interventional Radiology, University Medical Centre, Göttingen, Germany; 4 Institute for Diagnostic and Interventional Radiology, Klinikum St. Marien Amberg, Amberg, Germany; 5 Department for Diagnostic and Interventional Radiology, University Hospital Heidelberg, Heidelberg, Germany; 6 TLRC (Translational Lung Research Center), University Hospital Heidelberg, Heidelberg, Germany; 7 EPflex Feinwerktechnik GmbH, Dettingen an der Erms, Germany; 8 DZHK (German Centre for Cardiovascular Research), Partner Site Göttingen, Göttingen, Germany; 9 Institute of Biomedical Imaging, Graz University of Technology, Graz, Austria; 10 Laboratory Animal Science Unit, Leibniz-Institut Für Primatenforschung, Deutsches Primatenzentrum GmbH, Göttingen, Germany; 11 Department of Neuropathology, University Medical Centre, Göttingen, Germany; 12 Department of Cardiology and Pneumology, University Medical Centre, Göttingen, Germany

**Keywords:** Biopsy, Magnetic resonance imaging (interventional), Synchrotrons, X-ray microtomography

## Abstract

**Background:**

Diagnostic accuracy of endomyocardial biopsy could improve if clinically safe magnetic resonance (MR)-compatible bioptomes were available. We explored two novel MR-compatible cardiac bioptomes for performance, safety, and clinical viability, employing *in vivo* minipig trials and phase-contrast synchrotron radiation computed microtomography (SRµCT).

**Methods:**

Analysis of *ex vivo* obtained pig endomyocardial biopsies was performed using phase-contrast SRµCT and conventional two-dimensional histology. The technical performance was evaluated by measuring volume, inner and outer integrities, compression, and histological diagnostic value in 3 sets (6 per set) of biopsies for each experimental bioptome. The bioptomes were tested *in vivo* in 3 healthy minipigs per bioptome. The clinical feasibility was evaluated by procedural and cutting success as well as histological diagnostic value.

**Results:**

The bioptome with the ‘grind-grind’ design achieved similar values to control in compression (*p* = 0.822), inner (*p* = 0.628), and outer (*p* = 0.507), integrities *ex vivo*. It showed a better performance in the *in vivo* real-time MRI setting demonstrating a higher cutting success (91.7%) than the ‘grind-anvil’ (86.2%) design. In both *ex vivo* and *in vivo* evaluations, the ‘grind-grind’ design displayed sufficient diagnostic value (83% and 95%). The ‘grind-anvil’ design showed adequate diagnostic value both *ex vivo* and *in vivo* (78% and 87.5%) but was not comparable to control according to the three-dimensional (3D) analysis.

**Conclusion:**

A novel MR-compatible bioptome was identified as plausible in a clinical setting. Additionally, SRµCT and subsequent 3D structural analysis could be valuable in the label-free investigation of myocardial tissue at a micrometer level.

**Relevance statement:**

Implementation of MR-guided biopsy can improve animal studies on structural myocardial changes at any point in an experimental setup. With further improvements in guiding catheters, MR-guided biopsy, using the new bioptome, has a potential to increase quality and diagnostic accuracy in patients both with structural and inflammatory cardiomyopathies.

**Key points:**

• Novel MR-compatible bioptomes show promise for a clinical application.

• SRµCT enabled detailed analysis of endomyocardial biopsies.

• The bioptomes showed adequate *in vivo* performance without major complications.

**Graphical Abstract:**

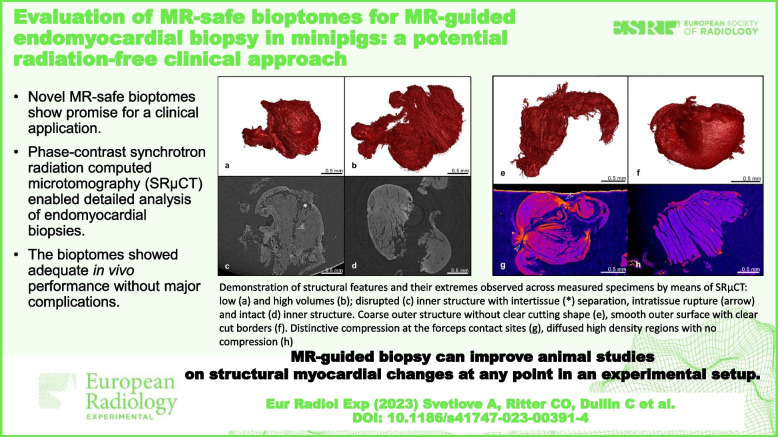

**Supplementary Information:**

The online version contains supplementary material available at 10.1186/s41747-023-00391-4.

## Background

Endomyocardial biopsy (EMB) provides diagnostic information in acute myocarditis, chronic inflammatory cardiac diseases, arrhythmias, tumours and storage diseases. It is frequently utilised in patients with unknown causes of heart failure and cardiomyopathies as well as in monitoring cardiac transplant success evaluation and rejection [1]. EMB is routinely performed under fluoroscopic guidance in the catheterisation lab and, while refined, has remained conceptually unchanged since its invention [2–5]. Due to its invasive nature indications for EMB have decreased over time [6]. Nevertheless, skilled intervention has a low procedural risk and major complication risks of less than 1% and provides unique information in the diagnostic workup [7, 8].

EMB is recommended in patients presenting with heart failure where a more precise diagnosis may influence the choice of therapy; specifically in patients that additionally present with hemodynamic challenges, dilated left ventricle and high-grade arrhythmias [9]. When successful, EMB is diagnostically invaluable; however, the primary drawback of the procedure is poor diagnostic yield [9]. Diagnostic success rate of the procedure is limited since planar radiographs used in fluorography offer no contrast in soft tissue [10]. The sampling is done mostly blindly without any knowledge of the location of the affected myocardium; it is therefore prone to error in focal, multifocal and localised disease patterns. A minimum of 5 samples is typically obtained in a single ventricle EMB. While this number is linked to 98% sensitivity to cardiac allograft rejection, the diagnostic yield in acute myocarditis varies within 21–78% success rate in single or bi-ventricular biopsy [1, 7, 10]. A secondary challenge is the operation of a bioptome and biopsy extraction from a defined region under persistent cardiac motion. Finally, post-transplant patients undergo a series of biopsies for routine control [11]. Additionally, it is recommended that an EMB operator maintains their procedural skill by performing a minimum of 50 biopsies a year [12]. Therefore, radiation exposure is repeated for both patients and physicians.

Alternative guidance methods such as electroanatomic mapping, cardiac magnetic resonance (CMR) and their combinations have been explored [13–16]. CMR imaging is one of the basic diagnostic tools in the diagnostic workup of myocarditis and other myocardial diseases affecting function and/or tissue integrity [17–19]. Thus, endomyocardial biopsy (EMB) under CMR guidance is a promising technique. In a proof-of-concept study, we have previously shown that real-time CMR-guided EMB performed in induced left ventricle focal high-frequency ablation lesions had a higher diagnostic success rate than standard fluorographic-guided EMB [20]. Nevertheless, magnetic resonance (MR)-compatible bioptomes are still not commercially available and are yet to be translated into the clinical setting, while conventional clinical bioptomes cannot be used due to paramagnetic materials in their composition.

A systematic comprehensive evaluation of how such bioptomes interact with tissue is required before safe clinical translation is considered. Phase-contrast synchrotron radiation computed microtomography (SRµCT) is a powerful tool for non-destructive high-resolution assessment of soft tissue [21–24]. *Ex vivo* phase-contrast SRµCT imaging can be performed on obtained biopsies, allowing label-free three-dimensional (3D) structural analysis of the tissue. This study discusses the development and modifications of the bioptome forceps design, narrowing down to two candidate solutions. Our aim was to quantify the degree of integrity of the biopsied tissue structure using SRµCT imaging as well as evaluate the performance of the bioptomes in *in vivo*.

## Methods

### Bioptomes

The forceps prototypes and the corresponding materials were designed by EPflex (Dettingen an der Erms, Germany). The designs featured variable non-magnetic materials, cutting mechanisms, opening angles, shaft mechanics and MR passive markers. In total nine different iterations were tested in explanted porcine heart tissue (data not shown). The two most promising bioptome iterations were used for further experiments described in this work.

### *Ex vivo* evaluation and sampling

The exploratory *ex vivo* procedure was performed in a freshly explanted porcine heart to test for two features: mechanical stability and cutting success. To simulate the *in vivo* procedure, the left ventricle was opened via a cut along the left anterior artery, and the bioptomes were pulled against the endocardial border of the left ventricular wall at a 70°–90° angle (visible control) while the heart was held in position by hand.

Selected two bioptome candidates (B1, B2) and a commercially available standard steel 5.5 F bioptome (CN, Cordis Biopsy Forceps, Johnson & Johnson, Miami Lakes, FL, USA) were further tested in a defined *ex vivo* experimental setting. The experimental setting was composed of a specially designed fixture for mounting the heart and a jig guiding the bioptome for controlled and replicable sample taking. In the guiding jig, firstly, the forceps were opened to the maximum opening angle and then pressed perpendicularly with constant force against the endocardium. Secondly, the forceps were closed via a pulling wire with constant pulling force. The bioptome cut into the tissue and the constant pulling force resulted in the retraction of the bioptome until the samples were detached from the heart. To mimic the clinical setting, six consecutive samples were cut with the same forceps, repeating the set three times. The CN bioptome was used in a single set of six biopsies.

### Sample preparation

The samples were placed in 10% buffered formalin solution for at least 24 h upon collection. They were then subjected to a standard histopathological tissue processing protocol with automated ascending ethanol series (60% 70%, 90%, 100%, xylene, each 1.5 h; dehydration automat, Suesse Labortechnik, Gudensberg, Germany) and subsequent paraffin embedding. An 8-mm diameter paraffin cylinder containing the tissue was extracted from the paraffin block by a clinical biopsy punch (PFM Medical, Cologne, Germany).

### Phase-contrast SRµCT imaging protocol and data segmentation

The cylinders were imaged at the SYRMEP (SYnchrotron Radiation for MEdical Physics, Trieste, Italy) beamline of the Italian synchrotron ELETTRA (Trieste, Italy). Access to the imaging node was provided by Euro-Bioimaging [25]. High-resolution SRµCT scans were obtained using the white beam setup (22 keV) with 2 µm voxel size and sample-to-detector distance of 9 cm. In total 3,600 projections were acquired in a 360° scan with an exposure time of 20 ms. The 3D SRµCT data sets were reconstructed using SYRMEP Tomo Project (STP Version 1.6.3). The phase retrieval was performed using TIE-Hom—a single distance algorithm by Paganin et al. [26] with a delta-to-beta ratio of 300. Following SRµCT scans, the 8-mm cylinders were re-embedded in paraffin blocks for further histological processing.

The 3D µCT datasets were rendered using VG Studio Max version 3.2.1 (Heidelberg, Germany). Biopsies were individually segmented using a region-growing algorithm. The segmentation was performed by a scientist with 4 years of experience in SRµCT and image processing. All subsequent computation was performed using a custom script written in Python 3. From the segmentation, the total number of voxels per biopsy was derived. The volume was calculated by multiplying the total number of voxels with the voxel size of 2 × 2 × 2 µm^3^. The compression feature was evaluated with a scoring procedure in a blinded manner with 3 µCT experts (with 4, 7, and 12 years of experience in the field) by reviewing each entire specimen in 3D. The scoring protocol was designed to evaluate the presence and severity of the compression due to dull cutting. The following scale was adopted: 0, no increased density regions, or diffused high density at the cutting position, indicating no or minimal compression; 1, definitive single pinching site with notable high density, indicating moderate compression; and 2, definitive one or two pinching sites with deformation and high density, indicating severe compression. The median score between the three observers was used.

Inner integrity was defined as the ratio between the segmented volume of the tissue and the volume calculated after inner rupture filling. First, a morphologic ‘closing’ procedure was performed in 3D with a single iteration using a 3 × 3 × 3 kernel to virtually fill the small cracks inside the specimen. It was followed by a morphologic ‘filling holes’ procedure that filled the remaining voids in the inner tissue. The resulting volume therefore lacks inner ruptures, that do not protrude to the outside surface. The inner integrity measure approaches 1 with higher integrity. The outer integrity was defined as the ratio between the filled segmented volume and the surface smoothed volume. To stop the inner cracks of the tissue from contributing to the calculation, ‘filling holes’ was performed to repair the cracks that do not protrude to the surface. The surface smoothing procedure was then performed through morphologic ‘opening’ with 3 × 3 × 3 kernel in a single iteration. The outer integrity measure approaches 1 with a higher quality of the specimen.

### *In vivo* evaluation

Six female minipigs of the Göttingen strain [27] weighing 30–46 kg and aged 12–24 months were sedated and put under anaesthesia, subsequent oral intubation and ventilation as well as mediation were performed as previously described [20]. The right or left femoral artery was punctured and an 8.5-F deflectable guiding catheter with a 0.035-inch MR-compatible guidewire (MaRVis Interventional, Hannover, Germany) was introduced into the left ventricle by a retrograde approach.

The animal was placed into a 3-T clinical MR scanner (Skyra, Siemens Healthineers, Erlangen, Germany, cardiac 18-channel body array coil). The imaging and biopsy procedures were performed as previously described [20]. Briefly, function images were obtained with standard balanced steady-state free precession sequences along standard views (three long-axis views and a stack of short-axis views) followed by real-time movies using radial two-dimensional fast low angle shot with non-linear inverse reconstruction [28, 29]. The bioptome was guided under real-time visual control (passive tracking) and interactive adjustment of the imaging plane. The interventionalist could look at the MR images online on a dedicated in-room monitor located near the animal table. Navigation could be achieved by interactively changing the scan plane using the user interface of the MR system. The interventionalist and operator at the console of the MR scanner communicated using an integrated MR-compatible communication system. Papillary muscles, aortic valve as well as mitral valve served as landmarks. The bioptome position was adjusted by keeping the tip of the device in the plane and directing it to the wall area. At least six biopsies were obtained from each procedure. After the biopsy, the animals recovered from anaesthesia under veterinarian observation, with further rest in their boxes. The animals were kept in the experimental animal unit of the University Medical Centre Göttingen.

Procedural success and cutting success of the *in vivo* experiments were evaluated. Procedural success was considered to be positive if at least six endomyocardial biopsies were obtained from a single animal without any major complications such as hemodynamically relevant pericardial effusion or death. The cutting success rate was defined as the number of acquired endomyocardial biopsies per number of cutting manoeuvres consisting of opening and closing of the bioptome at the assumed endocardial position.

To ensure the safety of the animal, after the biopsy procedure, CMR sequences for cardiac function and detection of pericardial effusion were taken. Images for function were obtained with standard balanced steady-state free precession sequences in three long-axis views and a stack of short-axis views after biopsy recovery. In case of a haemorrhage, a mass would be seen in the pericardial sac. In addition, late gadolinium enhancement was performed in all animals using 1 mmol/kg body weight of gadobutrol (Gd-BT-DO3A, Gadovist®, Bayer Schering AG, Leverkusen, Germany) injected intravenously.

All animal protocols were reviewed and approved by the local animal ethics committee as well as the governmental animal care and use committee (Bezirksregierung Braunschweig, Germany).

### Histological evaluation

Paraffin-embedded samples were sectioned into 2-µm slices (*ex vivo* obtained) or 5-µm slices (*in vivo* obtained) using a standard microtome (HM 340 E microtome, Thermo Fischer, Scientific,Waltham, MA, USA). The sections were deparaffinised at 60° and rehydrated through descending ethanol series (xylene, isopropanol, 98%, 75%, 60% EtOH, H_2_O; each 5 min). Histochemical staining with haematoxylin and eosin was performed on the sections [30]. All histological slides were reviewed in a blinded manner by two trained physicians, a pathologist with more than 20 years of experience and a cardiologist with more than 25 years of experience in evaluation of cardiac specimens, and scored for diagnostic value. The biopsies were rated in a binary manner by both of the physicians at the same time with a reached agreement. The designated criteria were either good diagnostic quality (Supplementary Figure S1a) with non-lacerated myocardium or minor/non-diagnostic quality with deformation damage and too few cellular units (Supplementary Figure S1b).

### Statistical analysis

The biopsies belonging to three sets in the *ex vivo* experiment showed no variability between the sets in the SRµCT based analysis (Supplementary Figure S2), therefore the sets were pooled for further comparison. Statistical analysis and representation were performed in GraphPad Prism Version 9.5.1 (Dotmatics, Boston, MA, USA). Statistical difference between an experimental bioptome and the control device was determined using a Mann–Whitney *U* test, assuming non-Gaussian distribution with *p* < 0.05 difference cut-off value.

## Results

### Bioptome design: narrowing down the solution

Conceptually, the forceps design mimics the clinically used forceps, featuring two half-cylindrical ‘jaws’ that grip the sample during closing. In the exploratory *ex vivo* phase the first three prototype iterations, produced with resin as a non-magnetic material, were found to have insufficient cardiac wall contact and insufficient cutting force. Further, the connections between the bioptome body and the forceps were disrupted during the process. Thus, these prototypes were not considered for further evaluation. The next two iterations were produced with titanium-based forceps, which resulted in improved cutting results. Depending on the angle and contact force, 70–90% cutting success (successful cut with the specimen in forceps/cutting trial) was achieved. However, the connection between the forceps and the body was disrupted after two or three collection attempts due to the instability of the adhesive material, resulting in a complete defect of the bioptome.

**Fig. 1:**
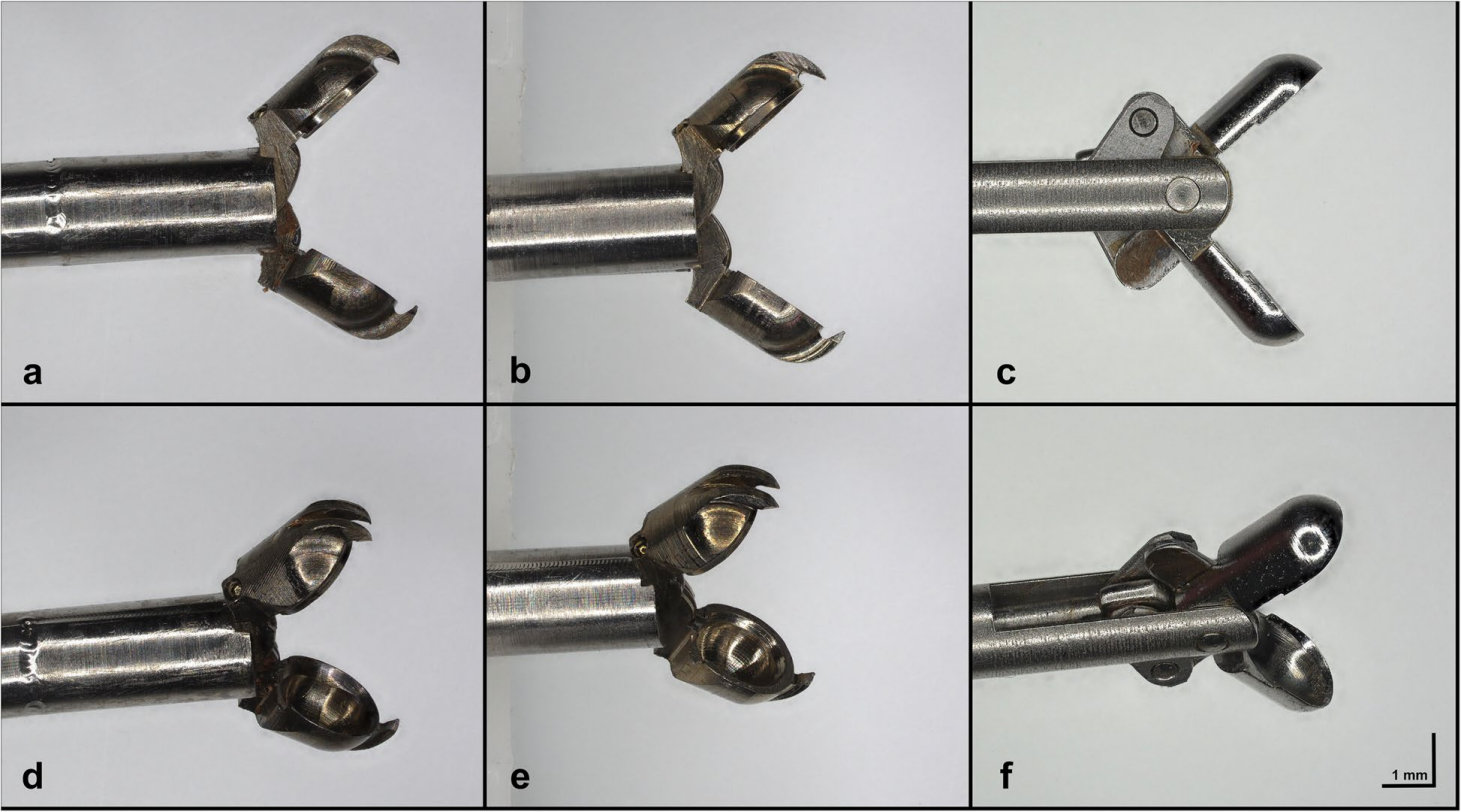
Novel magnetic-resonance-compatible bioptomes. Open side views of B1 (**a**), B2 (**b**) and the conventional clinical bioptome (**c**). Tilted open-view demonstration of the difference in the inner configuration of the forceps with “grind-grind” design (**d**) and “grind-anvil” design (**e**) and the conventional clinical design (**f**)

To further improve contact grip, the ‘jaws’ of the forceps were sharpened, and gripping bolts were incorporated at the tips. The diameter of the forceps was reduced from 2.3 mm to 2.0 mm to bring the procedure closer to the clinical setting. Since the opening angle is a major contributor to the cutting success, it was further widened to a technically limited maximum of 78°. Moreover, the mechanical strain in the *in vivo* setting was expected to be more challenging compared to *ex vivo* tests. Pulling the bioptome back into the catheter exerts mechanical stress on the connection between forceps and the bioptome body. Thus, further improvement of the body-forceps connection was necessary and accomplished in the iterations finally used. Following these modifications, two prototypes were chosen for further testing (Fig. 1), with the first one “B1” (Fig. 1a, d) having 2 sharpened ‘jaws’ (grind-grind) and the second “B2” (Fig. 1b, e) having one sharpened and one unsharpened jaw (grind-anvil). All other mechanical features of the forceps were comparable in both iterations.

### Structural analysis of *ex vivo* biopsies with SRµCT imaging

**Fig. 2:**
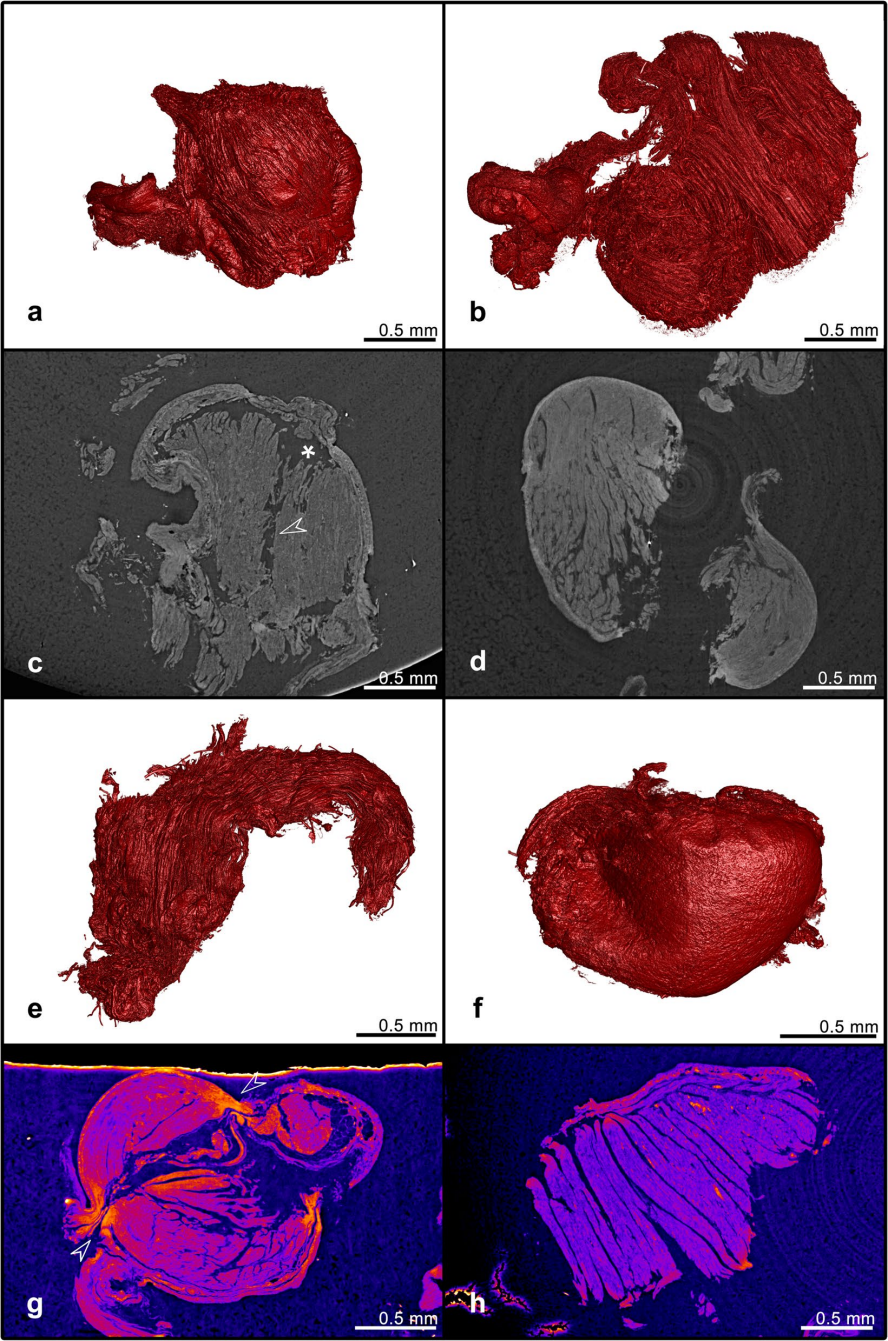
Demonstration of structural features and their extremes observed across measured specimens by means of synchrotron radiation computed microtomography. Low (**a**) and high volumes (**b**). Disrupted (**c**) inner structure with intertissue (*) separation, intratissue rupture (arrow) and intact (**d**) inner structure. Coarse outer structure without clear cutting shape (**e**), smooth outer surface with clear cut borders (**f**). Distinctive compression at the forceps contact sites (**g**), diffused high density regions with no compression (**h**)

SRµCT analysis was performed on *ex vivo* obtained biopsies to understand the effect of the bioptome on the sampled tissue. Four main features were identified and evaluated: tissue volume, inner tissue integrity, outer surface integrity and presence of compression sites. Figure 2 shows example extremes of the outlined features. The variability in volume is displayed with a smaller specimen (a, 0.3232 mm^3^) and a larger example (b, 1.6287 mm^3^). Inner structure rupture can be observed in Fig. 2c, with intertissue interface parting (star) and intratissue haemorrhage (arrow). An example of regular inner myocardial striations due to anatomy is shown in Fig. 2d. Outer tissue integrity variability is seen in examples Fig. 2e, f; the specimen in Fig. 2e displays a coarse uneven outer surface compared to the smooth rounded shape of the specimen in Fig. 2f. Procedural compression due to tissue–bioptome interaction was another prominent feature. Figure 2g shows two distinct pinches at the opposing sides (arrows) with increased tissue density indicating a dull cut, while Fig. 2h shows a cleaner cut with high-density regions but no obvious pinching.

**Fig. 3:**
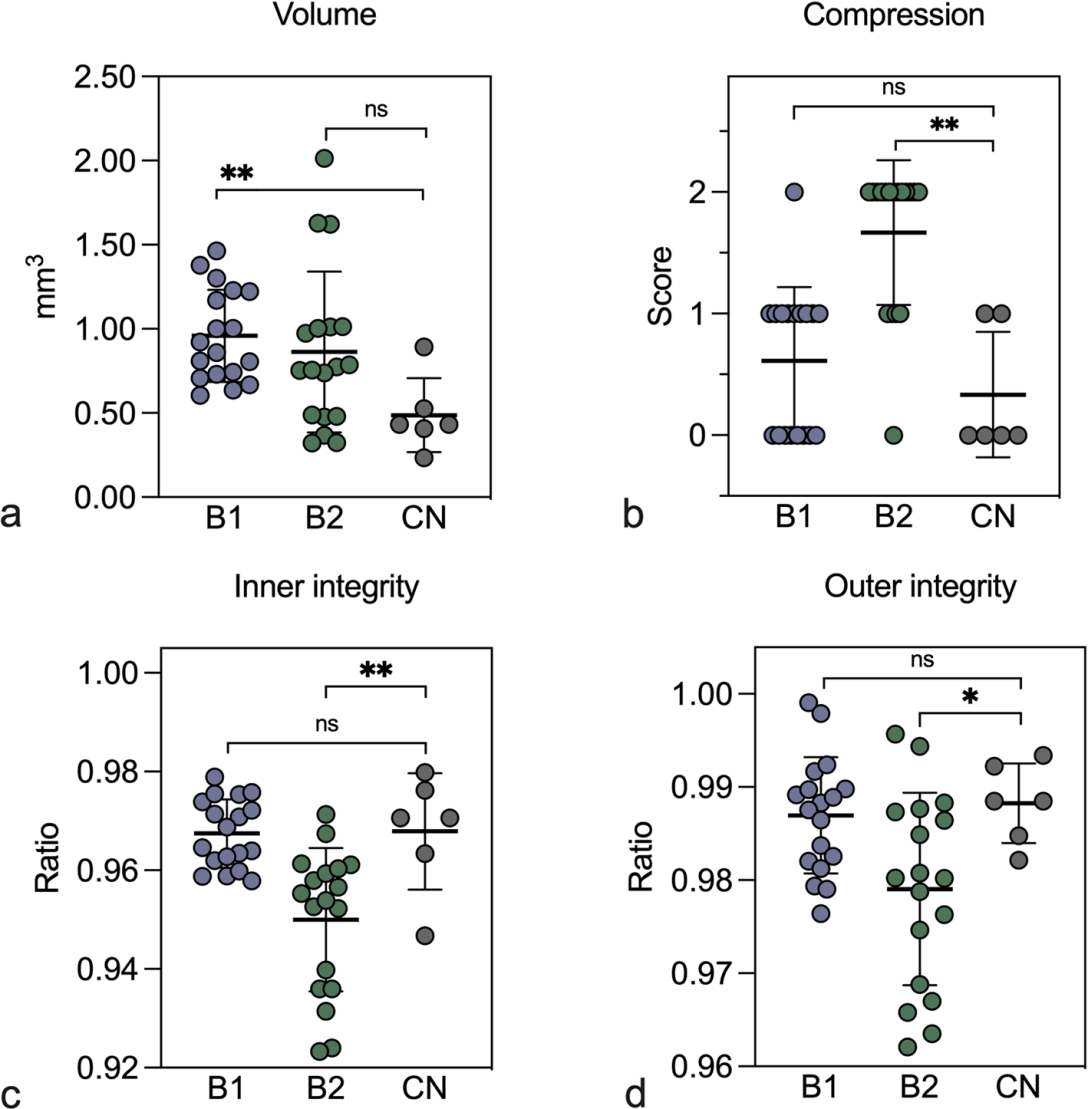
Synchrotron radiation computed microtomography-based analysis of the notable structural features. **a** B1 acquired specimens demonstrate a significantly higher volume compared to control, a large variability in volumes can be noted in all tested bioptomes (**p = 0.0013). **b** B2 displays a significantly higher compression score than the control bioptome while B1 scores comparably to control (**p = 0.0011). **c** Inner integrity ratio estimates the amount of rupture inside the biopsied tissue (approaching 1.00 = less rupture), the structure of the of B1 samples was comparable to the control samples, while B2 obtained samples show a comparably low integrity (**p = 0.0091). **d** Outer integrity ratio estimates the amount of surface roughness on the biopsy, produced by the expulsion of the bioptome after the cut was performed (approaching 1.00 = less rupture). B1 specimens show a comparable outer integrity to the control biopsies while B2 has significantly lower outer integrity (*p = 0.0320). Statistical significance was determined by Mann–Whitney *U* test, assuming non-Gaussian distribution, *n* = 18, 18, and 6 for B1, B2, and control samples

Figure 3 shows the distribution of the tissue volume, inner tissue integrity, outer surface integrity and the compression scores. Bioptome B1 produced larger specimens compared to the controls while the volumes of B2 obtained specimens did not differ from the controls (Fig. 3a). The biopsies produced by B1 showed a comparable compression score to the control bioptome (Fig. 3b). Biopsies produced by B2 scored higher on average than the control, indicating a higher degree of tissue crushing. The inner structure of the specimens produced by B1 was not significantly different from the control specimens, as measured by the inner integrity ratio (Fig. 3c). Furthermore, specimens produced by B1 showed no significant difference in the outer integrity ratio to those produced by the control (Fig. 3d), indicating that the pressure exerted on the explanted tissue by B1 is comparable to that of the conventional steel bioptome. B2 specimen had significantly larger inner and outer rupture compared to the control as indicated by lower integrity ratios (Fig. 3c, d).

Following the SRµCT analysis, all specimens were sectioned and stained to recapitulate the standard diagnostic workup. The experimental bioptomes achieved similar scores to each other and to the control bioptome. Specimens produced by B1 were scored diagnostically valuable 83% of the time while those produced by B2 78% compared to the control specimens with 83%.

### *In vivo* performance

Table 1 shows the summary of the performance of the experimental bioptomes in the *in vivo* setting. Procedural success was measured at 100% with 3 animals per bioptome. The cutting success was marginally higher for B1 than for B2, similarly, the diagnostic value was higher in B1 obtained specimens than in B2.

**Table 1:** Results of the *in vivo* procedures performed with B1 and B2 experimental forceps. Three mini-pigs were used for each experimental bioptome with at least 6 specimens obtained per pig

Bioptome	Procedural success (%)	Cutting success (%)	Diagnostic value (%)
B1	100.0 ± 0.0	91.7 ± 7	95.3 ± 8
B2	100.0 ± 0.0	86.2 ± 4	87.5 ± 1

Data are given as mean ± standard deviation

**Fig. 4:**
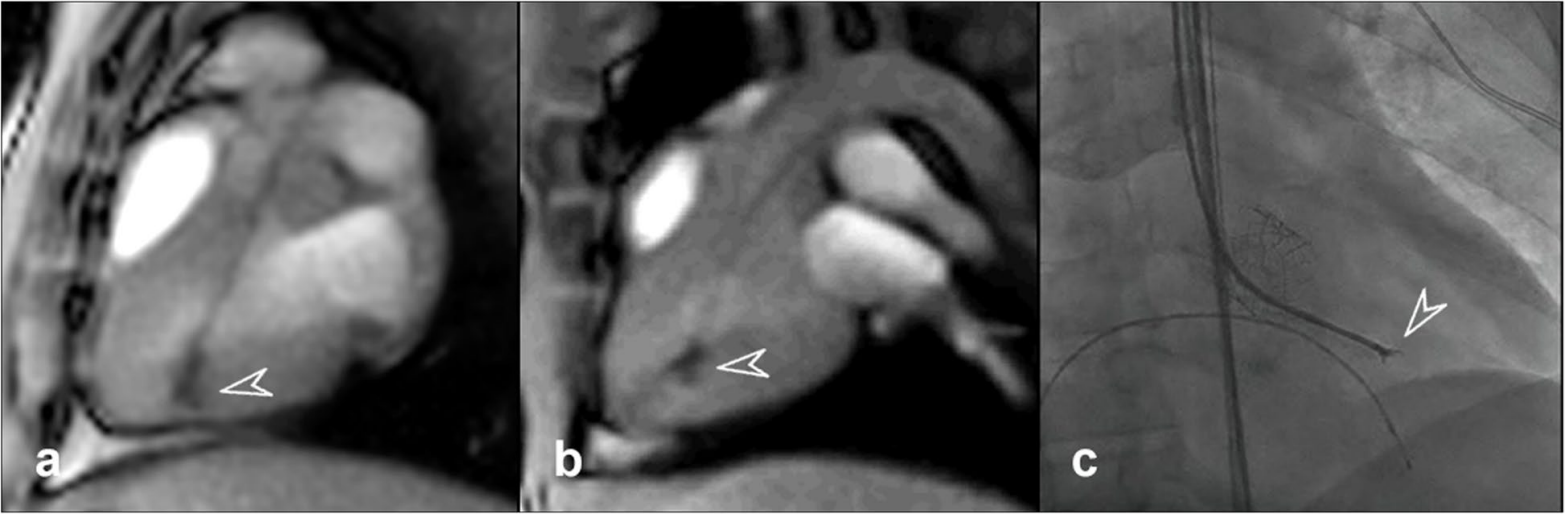
Real-time magnetic resonance imaging-based endomyocardial biopsy procedure depicting the “open” state during the *in vivo* procedure with B1 (**a**) and B2 (**b**), featuring visible cardiac walls, major vessels and the timing of the cardiac cycle. X-ray fluorography-based standard procedure in a patient showing an ‘open’ state control forceps (**c**)

Visibility of the bioptomes during the procedure was also evaluated. Figure 4 demonstrates the B1 (Fig. 4a), B2 (Fig. 4b) bioptomes during the real-time MRI-guided EMB procedure. A clear cardiac wall outline is visible with the forceps head positioned in the ventricle identified by the MR passive markers embedded in the shaft of both B1 and B2. Discerning all the states of the ‘open’ conformation appeared difficult during the procedures, while the end-open state can be recognised when the forceps are positioned mid-lumen (Fig. 4b, arrow), when pushed against the myocardium wall the visibility reduces (Fig. 4a, arrow). Comparatively, the standard EMB procedure demonstrates better visibility of all bioptome components (Fig. 4c). An example video demonstrating the use of B2 can be viewed in Supplementary Materials (Video S1).

Following the procedure, no major pericardial effusion was detected and visually no regional contraction abnormalities were observed. Additionally, no complications were observed in the recovery time.

## Discussion

This paper outlines the comprehensive analysis of two new MR-compatible bioptome candidates and their development. In the presented research two experimental bioptomes were tested *ex vivo* and *in vivo*. To that end, SRµCT analysis on the *ex vivo* obtained samples was performed to examine the volume and presence of structural abnormalities in the inner tissue and the outer surface.

The analysis revealed a large deviation in the measured specimen volumes in all of the examined groups. B1, compared to the control, was found to have a larger volume on average. Further, no difference in the inner rupture ratio between the B1 and the tested control was detected, while B2 score was significantly lower. The outer surface appeared to differ between B2 and the control, with B2 displaying a coarser outer surface. Finally, compression sites, appearing likely due to the impact of the bioptome, identify B1 as a less intrusive tool with higher preserving power. The compression sites observed have been reported previously as “crush artefacts”. These are difficult features to recognise in two-dimensional histological sections but are clearly evident in 3D SRµCT volumes. Depending on their severity, the crush artefacts may prove diagnostic analysis difficult or impossible [10, 31, 32]. The histological slices of all *ex vivo* biopsies were tested for diagnostic value; primarily the size and integrity of the myocardium were examined. The control samples were found to be diagnostically viable in 83% of the cases, B1 and B2 scored 83% and 78% respectively. Together these results indicate that despite structural differences, quantified by the SRµCT, between the B1, B2 and the control, the experimental bioptomes perform on par with the control device *ex vivo* and provide sufficient quality samples for diagnostic use. However, the observed clear differences in the structure of the obtained tissue indicate that the sight from which the biopsy was obtained may be more affected in the case of B2. To our knowledge, this is the first publication showing 3D quality assessment of cardiac biopsies obtained by MR-compatible bioptomes. There have been previous investigations into potentially translatable CMR bioptomes, which based their quality assessment on histology and visual analysis alone or in combination with histology [20, 33–35]. The size of the biopsy, an important factor in the diagnostic outcome, was typically measured by a ruler or from a histological slice (example: Figure S2). Based on the presented results we believe some of the finer differences cannot be effectively assessed by histology alone. SRµCT has proven to be a valuable tool in the measurement of accurate volumes and structural differences. We believe it should, in the future, be considered as an essential part of the safety pre-screening process before potential patient translation.

We further examined the performance of the bioptomes *in vivo.* Both of the bioptomes performed excellently under *in vivo* conditions with a 100% procedural success rate in all animals. Nevertheless, in line with the previous reports, the visualisation of the bioptome under MR conditions was challenging [20, 35], while the bioptome itself was clearly visible discerning the open and closed states of the forceps was difficult. Differentiation of the states was only possible due to the changes in the artefact contours and its surroundings during continuous motion. In our experiments the CMR safe forceps diameter amounted to 2.0 mm. While the outer diameter of a standard guiding catheter should not exceed 7 F, this diameter is too small for a forceps diameter of 2.0 mm. The minimal forceps diameter of 1.97 mm allows a 7.5-F guiding catheter, an acceptable but not ideal size for the entire procedure. Therefore, the size of the forceps will be addressed in the future. Furthermore, targeted biopsy is still dependent on a catheter system that allows exact positioning of a bioptome, yet deflectable MR-safe catheters are currently not commercially available.

We believe that once the discussed concerns about catheter availability are addressed, the new proposed experimental forceps and detailed analysis including SRµCT are feasible and safe in larger animal experimental settings. Translation of MR-guided EMB procedure into clinical practice could reduce the number of required specimens and procedures by providing real-time superior cardiac anatomy visualisation to the operator. This in turn would reduce the risk of complications such as cardiac perforation and tamponade as well as repeated ionising radiation exposure. Additionally, advanced MRI sequences allow for pathological tissue characterisation of abnormal myocardium, in turn increasing the sampling probability in the diseased area. The resulting increased diagnostic yield in cases of myocarditis, infiltrative heart diseases and heart failure of unknown aetiology could significantly improve the precision of the diagnosis and offer individual therapeutic approaches with better outcomes.

Some limitations of this study should be taken into account. The entire MR-guided EMB procedure was slower than an x-ray-guided EMB and was overall more expensive. 3D SRµCT analysis relies on access to a synchrotron facility and large data processing power. The number of animals used in this study was small and the entire EMB as an MR-safe procedure has to be validated in a larger study. Additionally, while at this time no lesion targeting was performed, it should be tested once the remaining challenges are addressed. Therefore, the method is presently limited to experimental conditions and larger numbers of safe, successful biopsies have to be performed before a clinical application is considered.

In conclusion, the novel MR-compatible bioptomes are feasible in a large animal real-time MR-guided EMB procedure. A 3D volume analysis using SRµCT can be used to evaluate the technical performance of the bioptomes and the structural integrity of the tissue; subsequently, the tissue can be evaluated according to the standard pathological protocol. We determined that at least one bioptome model performed comparably to the clinically used control bioptome.

### Supplementary Information

**Additional file 1: Figure S1.** Histological H&E sections from *ex vivo* obtained biopsies. (A) shows a large tissue fragment featuring non-lacerated, non-deformed myocardium judged of sufficient diagnostic value. (B) example section of a non-diagnostically valuable sample with significant rupture and deformation. **Figure S2.** Distributions of SRµCT quantified values between the 3 consecutive sampling sets in B1 and B2. **Figure S3.** Bioptome B2 with typical myocardial specimen rated as “normal”. (A) specimen in the open forceps; (B) specimen in comparison to a beveled 18 Gauge (1.27 mm) Intradyn® introducer needle (Braun Melsungen, Germany).

**Additional file 2: Video S1.** Bioptome B2 in the left ventricle is pushed against the inferolateral wall, here visible in the left ventricular outflow tract view in real-time fast low angle shot sequences. The supporting guiding catheter is drawn back from the left ventricle into the aorta ascending just above the aortic valve. Open and closed states of the bioptome are distinguishable solely during movement.

## Data Availability

The data and analysis scripts are available from the first and corresponding authors upon reasonable request.

## Abbreviations

3D: Three-dimensional
CMR: Cardiac magnetic resonance
EMB: Endomyocardial biopsy
MR: Magnetic resonance
MRI: Magnetic resonance imaging
SRµCT: Synchrotron radiation computed microtomography
