# Supplementary material for: Evaluation of MR-safe bioptomes for MR-guided endomyocardial biopsy in minipigs: a potential radiation-free clinical approach
Source: Eur Radiol Exp. 2023 Dec 5;7:76. doi: 10.1186/s41747-023-00391-4 (PMC10695907; doi:10.1186/s41747-023-00391-4)
Supplement: Supplementary file 1 — Additional file 1: Figure S1. Histological H&E sections from ex vivo obtained biopsies. (A) shows a large tissue fragment featuring non-lacerated, non-deformed myocardium judged of sufficient diagnostic value. (B) example section of a non-diagnostically valuable sample with significant rupture and deformation. Figure S2. Distributions of SRµCT quantified values between the 3 consecutive sampling sets in B1 and B2. Figure S3. Bioptome B2 with typical myocardial specimen rated as “normal”. (A) specimen in the open forceps; (B) specimen in comparison to a beveled 18 Gauge (1.27 mm) Intradyn® introducer needle (Braun Melsungen, Germany). [file 41747_2023_391_MOESM1_ESM.docx]

**Evaluation of MR-safe bioptomes for MR-guided endomyocardial biopsy in minipigs: a potential radiation-free clinical approach**

**ELECTRONIC SUPPLEMENTARY MATERIAL**


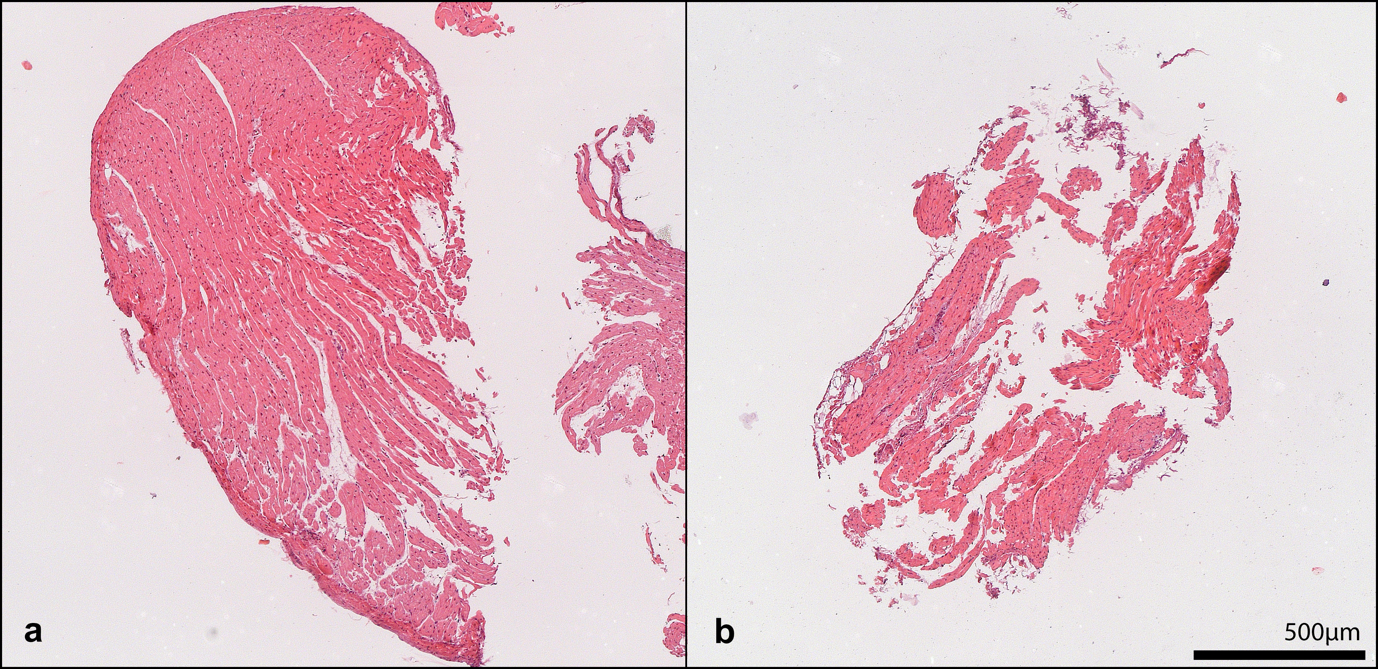


**Figure S1.** Histological H&E sections from ex vivo obtained biopsies. (A) shows a large tissue fragment featuring non-lacerated, non-deformed myocardium judged of sufficient diagnostic value. (B) example section of a non-diagnostically valuable sample with significant rupture and deformation.


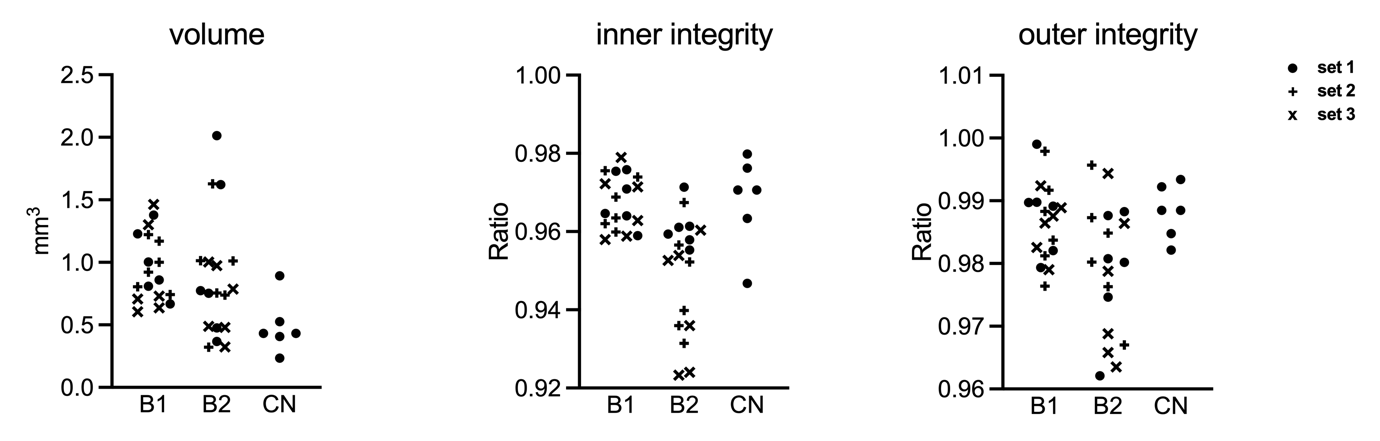


**Figure S2.** Distributions of SRµCT quantified values between the 3 consecutive sampling sets in B1 and B2.


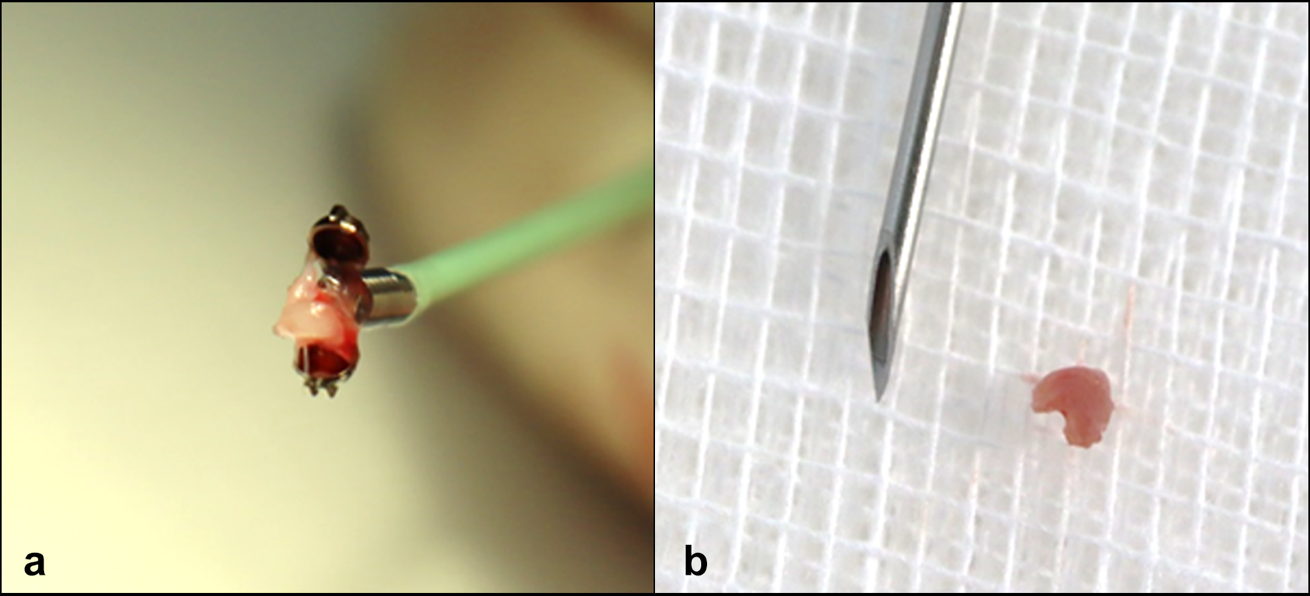


**Figure S3.** Bioptome B2 with typical myocardial specimen rated as “normal”. (A) specimen in the open forceps; (B) specimen in comparison to a beveled 18 Gauge (1.27 mm) Intradyn® introducer needle (Braun Melsungen, Germany).
